# Screening for Prostate Cancer by Digital Rectal Examination and PSA Determination in Senegal

**DOI:** 10.5402/2011/943704

**Published:** 2011-07-10

**Authors:** Lamine Niang, Charles N. Kouka, Mohamed Jalloh, Sérigne M. Gueye

**Affiliations:** Urology Service, Grand Yoff General Hospital, BP 3270 Dakar, Senegal

## Abstract

*Objectives*. The goal of our study was to investigate the prevalence of prostate cancer in an unselected population of Senegalese men. *Patients and Methods*. We conducted the study over two years (2008 and 2009) on an unselected population of 572 Senegalese men, aged 35 and older. The following parameters have been investigated: the subject's age, the presence or absence of urination disorders, the family's history of prostate cancer or prostate surgery, the aspects of the prostate on digital rectal examination (DRE), the total PSA level, and the outcomes of the prostate biopsies. Data entry was performed with Epi Info 6 software and was analyzed and recorded using Excel software. We performed mean and frequency calculations. *Results*. The mean age of our patients was 65.5 years, with extremes of 38 and 93 years. Age groups from 50 to 59 and from 60 to 69 were the most represented. DRE was normal in the age group from 35 to 39, and only one patient in the age group from 40 to 49 had a prostate nodule. PSA level was greater than or equal to 4 ng/mL in 66 cases. A total of 5.4% patients had a PSA level greater than or equal to 10 ng/mL. Only two patients in the age group from 40 to 49 had a PSA level greater than 4 ng/mL. Of the 72 biopsies we performed, prostatic adenocarcinoma was found in 30.6% of the cases. It is the only type of prostate cancer we found in our series. The cases of prostate cancer were mostly observed in the age groups from 60 to 69 and from 70 to 79. No cases were detected for ages younger than 50. DRE gave indications of possible adenocarcinoma in 27.30% cases. Its sensitivity was 27%, while its positive predictive value was estimated at 35%. Of all positive biopsies, 4.5% had a PSA level between 0 and 3.9 ng/mL. In this case, the sensitivity of PSA was 95.5%, and the positive predictive value was 31.8%. High-grade intraepithelial neoplasiae were observed in 21 cases. *Conclusion*. Prostate cancer is frequent in Senegal, and screening remains the best way for early diagnosis.

## 1. Introduction

The early detection and treatment of prostate cancer continues to pose multiple debates. Early detection of certain cancers allows for a higher, more productive treatment program. Detection is based on digital rectal examination (DRE) and the determination of prostate-specific antigen (PSA) levels. It is recommended to begin screening for detection of prostate cancer at the age of 50 in men with more than 10 years of life expectancy, and at the age of 45 in men with a familial history of prostate cancer [1]. While in developed countries, the mortality rate of prostate cancer has declined significantly thanks to early detection; in Africa, the diagnosis is most often delayed because patients only consult in cases of urinary disorders, resulting in 80% of cases being diagnosed in a metastatic state [2]. The aim of our study was thus to investigate the prevalence of prostate cancer in an unselected population of Senegalese men to propose an early detection system for localized forms. 

## 2. Patients and Methods

We conducted a prospective study, over two years (2008 and 2009), on an unselected Senegalese population of male patients. Our study included 572 patients whose age was greater than or equal to 35. We investigated the following parameters:

- the subject's age,
- the presence or absence of urination disorders,
- the family's history of prostate cancer or prostate surgery,
- the appearance of the prostate on digital rectal examination,
- the total PSA level,
- the outcome of the prostate's biopsy.

The data entry was performed with Epi Info 6 software. The data was analyzed and recorded using Excel software. We performed both mean and frequency calculations. 

## 3. Results

**Figure 1:**
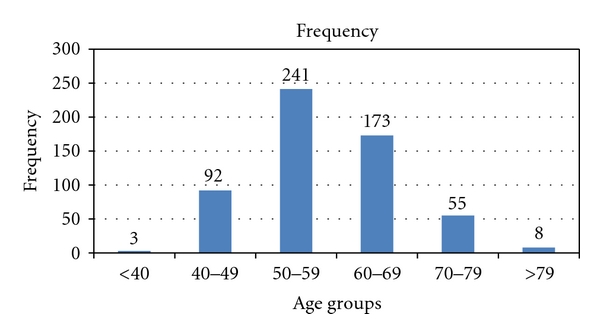
Distribution of patients by age.

The mean age of our patients was 65.5 years, with extremes of 38 and 93 years. Age groups from 50 to 59 and from 60 to 69 were the most highly represented.  Figure 1 shows the distribution of patients according to age groups.

**Figure 2:**
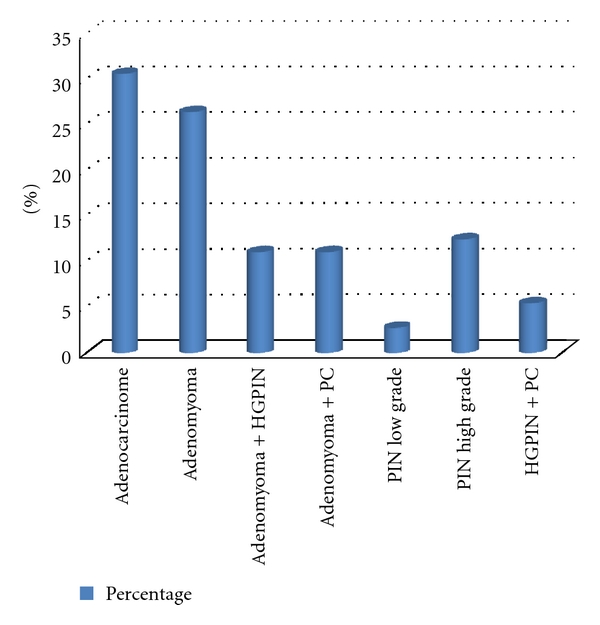
Results of prostate biopsies.

**Figure 3:**
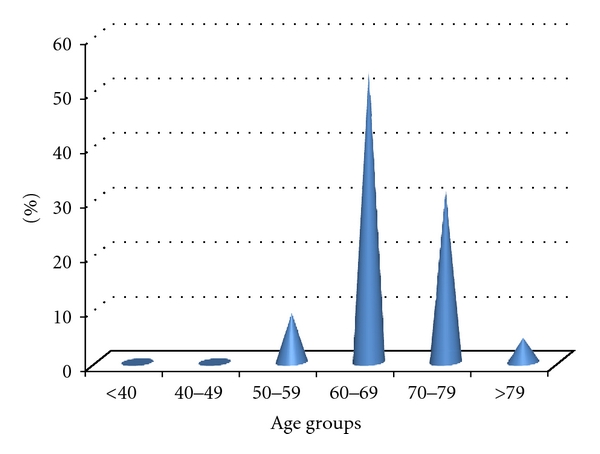
Distribution of adenocarcinoma by age.

The various aspects of the prostate on DRE are shown in Table 1. The prostate was benign in 54.9% of the cases. DRE was normal in the age group from 35 to 39, and only one patient in the age group from 40 to 49 had a prostate nodule. A total of 21.5% of participants had a PSA level greater than or equal to 4 ng/mL, which represented 66 cases. In addition, 5.4% of patients had a PSA greater than or equal to 10 ng/mL. Only two patients in the age group of 40 to 49 had a PSA level greater than 4 ng/mL. The distribution of PSA levels as a function of age is reported in Table 2. Of the 72 biopsies we performed, prostatic adenocarcinoma accounted for 30.6% of them. It is the only type of prostate cancer we found in our series. Other histological lesions were associated with prostate cancer (Figure 2). The distribution of histological lesions according to age groups is reported in Table 3. The cases of prostate cancer were mostly observed in the age groups from 60 to 69 and from 70 to 79. No case was detected for men younger than 50 (Figure 3). DRE was suspicious in 27.3% of cases. Its sensitivity was of 27%, while its positive predictive value was estimated as 35%. Four and one-half percent of all positive biopsies had PSA levels between 0 and 3.9 ng/mL. When PSA levels were above 30 ng/mL, the detection rate was 36.36%. The sensitivity of PSA levels was 95.5%, and the positive predictive value was 31.8%. High-grade intraepithelial neoplasiae were associated in 12 cases with adenomyoma injuries or chronic prostatitis lesions. However, they were only isolated in nine cases. These high-grade lesions were observed in 21 cases, of which the age group from 60 to 69 was the most represented. 

## 4. Comments

In our study population, the prevalence of prostate cancer was 3.8%. This prevalence is almost the same as observed in Korea [3]. In our study, the detection rate of adenocarcinoma on biopsy was 30.5%. Cosimo de Nunzio detected 30% of adenocarcinoma in a series of biopsies with 12 samples systematically collected [4]. Historically, DRE has been used as a method of early detection of prostate cancer. Any abnormal DRE requires the performance of a prostate biopsy, even if PSA levels are normal [5]. The detection rate of prostate cancer based on prostate biopsy when DRE is abnormal and PSA levels are lower than 4 ng/mL is variably reported in the literature, ranging from 3% to 41% [6, 7]. Furthermore, rectal examination is very operator dependent [8, 9]. Thus, digital examination of the prostate by DRE is a poorly reliable method, with a sensitivity lower than 50%. Normal examination does not eliminate the possibility of a cancer. However, when associated with PSA levels, its predictive value is increased, and thus our decision to use it as a screening tool in our study. This is also the case in other studies performed in Europe, Asia, and United States [1, 3]. The impact of isolated prostatic intraepithelial neoplasia (PIN) lesions on prostate biopsies is not clear and varies from 0.7 to 19%, depending on the population studied (general population versus population with urological pathologies), the degree of severity considered, and the country selected [10, 11]. In the study by Parkinson [12] and Wills et al. [13], 439 sets of sextant biopsies performed with an 18-gauge needle were reviewed, and the diagnosis of high-grade PIN was discovered in 5.5% of these series of biopsies. In the study by Bostwick, the incidence of isolated high-grade PIN was 16.5% [14]. In our study population, the PIN incidence was 29.1%. According to some authors, prostatic intraepithelial neoplasia lesions constitute precancerous lesions [11–16]. The prevalence of PIN lesions increases with age, and it appears that there is a five-year gap before cancer onset [17, 18]. 

## 5. Conclusion

Prostate cancer is diagnosed at advanced stage in our countries; screening must be recommended for early diagnosis and curative treatment. 

## Figures and Tables

**Table 1:** Aspect of the prostate on digital rectal examination depending on age.

Age	Normal	Hbp	Indurated	Nodular
<40	3	0	0	0
40–49	67	24	0	1
50–59	127	111	2	1
60–69	35	129	8	0
70–79	6	46	1	3
>79	3	4	0	1

Total	241	314	11	6

**Table 2:** PSA level distribution depending on age.

Age	0–3.9 ng/mL	4–9.9 ng/mL	10–19.9 ng/mL	20–29.9 ng/mL	>30 ng/mL
<39	3	0	0	0	0
40–49	90	1	1	0	0
50–59	228	12	0	0	1
60–69	140	18	7	5	3
70–79	40	3	5	0	7
>80	5	1	2	0	0

Total	506	35	15	5	11

**Table 3:** Distribution of histological lesions depending on age.

Age	Adenocarcinoma	Adenomyoma	Adenomyoma + high-grade PIN	Adenomyoma + chronic prostatitis	Low-grade PIN	High-grade PIN	High-grade PIN + chronic prostatitis
<40	0	0	0	0	0	0	0
40–49	0	1	0	1	0	0	0
50–59	2	5	1	1	0	3	2
60–69	12	9	5	5	2	3	1
70–79	7	2	2	1	0	3	1
>79	1	2	0	0	0	0	0

Total	22	19	8	8	2	9	4
